# An Italian survey on the use of T-tube in liver transplantation: old habits die hard!

**DOI:** 10.1007/s13304-021-01019-1

**Published:** 2021-04-01

**Authors:** Riccardo Pravisani, Paolo De Simone, Damiano Patrono, Andrea Lauterio, Matteo Cescon, Enrico Gringeri, Michele Colledan, Fabrizio Di Benedetto, Fabrizio di Francesco, Barbara Antonelli, Tommaso Maria Manzia, Amedeo Carraro, Marco Vivarelli, Enrico Regalia, Giovanni Vennarecci, Nicola Guglielmo, Manuela Cesaretti, Alfonso Wolfango Avolio, Maria Filippa Valentini, Quirino Lai, Umberto Baccarani

**Affiliations:** 1 Liver-Kidney Transplantation Unit, Department of Medicine, University of Udine, Udine, Italy; 2 Hepatobiliary Surgery and Liver Transplantation, University Hospital Pisa, Pisa, Italy; 3 General Surgery 2U, Liver Transplant Center, A.O.U. Città della Salute e della Scienza di Torino, University of Torino, Turin, Italy; 4 General Surgery and Abdominal Transplantation, ASST Grande Ospedale Metropolitano Niguarda, Milan, Italy; 5 General Surgery and Transplantation Unit, Department of Medical and Surgical Sciences, Azienda Ospedaliero-Universitaria-Policlinico S.Orsola-Malpighi, Bologna, Italy; 6 Hepatobiliary Surgery and Liver Transplantation Unit, University Hospital, Padua, Italy; 7 Chirurgia Generale 3, Trapianti Addominali, Ospedale Papa Giovanni XXIII, Bergamo, Italy; 8 Hepatopancreatobiliary Surgery and Liver Transplantation Unit, University of Modena and Reggio Emilia, Modena, Italy; 9 Department for the Treatment and Study of Abdominal Diseases and Abdominal Transplantation, IRCCS ISMETT-UPMC, Palermo, Italy; 10 General and Liver Transplant Surgery Unit, Fondazione IRCCS Ca’ Granda Ospedale Maggiore Policlinico, Milan, Italy; 11 HPB and Transplant Unit, Department of Surgery Science, University of Rome Tor Vergata, Rome, Italy; 12 General Surgery and Liver Transplant Unit, University Hospital of Verona, Verona, Italy; 13 HPB Surgery and Transplantation Unit, Department of Clinical and Experimental Medicine, Polytechnic University of Marche, Ancona, Italy; 14 HPB Surgery and Transplantation Unit, Istituto Nazionale Tumori, IRCCS, Milano, Italy; 15 Laproscopic, Hepatic, and Liver Transplant Unit, AORN A. Cardarelli, Naples, Italy; 16 Division of General Surgery and Liver Transplantation, S. Camillo Hospital, Rome, Italy; 17 Liver Transplant Unit, Department of General Surgery, Azienda Ospedaliera G. Brotzu, Cagliari, Italy; 18 General Surgery and Liver Transplantation Unit, Fondazione Policlinico Universitario Agostino Gemelli IRCCS, Rome, Italy; 19 General Surgery and Liver Transplantation Unit, Department of Emergency and Organ Transplantation, University of Bari, Bari, Italy; 20 General Surgery and Organ Transplantation Unit, Sapienza University of Rome, Rome, Italy

**Keywords:** T-tube, Biliary complications, Survey, Bile acids

## Abstract

There is enough clinical evidence that a T-tube use in biliary reconstruction at adult liver transplantation (LT) does not significantly modify the risk of biliary stricture/leak, and it may even sustain infective and metabolic complications. Thus, the policy on T-tube use has been globally changing, with progressive application of more restrictive selection criteria. However, there are no currently standardized indications in such change, and many LT Centers rely only on own experience and routine. A nation-wide survey was conducted among all the 20 Italian adult LT Centers to investigate the current policy on T-tube use. It was found that 20% of Centers completely discontinued the T-tube use, while 25% Centers used it routinely in all LT cases. The remaining 55% of Centers applied a selective policy, based on criteria of technical complexity of biliary reconstruction (72.7%), followed by low-quality graft (63.6%) and high-risk recipient (36.4%). A T-tube use > 50% of annual caseload was not associated with high-volume Center status (> 70 LT per year), an active pediatric or living-donor transplant program, or use of DCD grafts. Only 10/20 (50%) Centers identified T-tube as a potential risk factor for complications other than biliary stricture/leak. In these cases, the suspected pathogenic mechanism comprised bacterial colonization (70%), malabsorption (70%), interruption of the entero-hepatic bile-acid cycle (50%), biliary inflammation due to an indwelling catheter (40%) and gut microbiota changes (40%). In conclusion, the prevalence of T-tube use among the Italian LT Centers is still relatively high, compared to the European trend (33%), and the potential detrimental effect of T-tube, beyond biliary stricture/leak, seems to be somehow underestimated.

## Introduction

In deceased donor liver transplantation (DDLT), the inclusion criteria for donation have progressively been expanded in recent years [1]. Older donors, those with multiple comorbidities, donors after circulatory death (DCD), or grafts with significant steatosis have increasingly been accepted [1–3]. Such trend has been determined by the increasing organ demand of LT candidates, who in turn have been listed with progressively worse clinical conditions [1]. However, low-quality graft and high-risk recipients are associated with an inherent greater risk of post-transplant morbidity, such as early allograft dysfunction, vascular and biliary complications [2, 3]. T-tube drainage has traditionally been used in DDLT to monitor the bile quality and output as a direct marker of graft function and to get an easy radiologic access to the biliary tree, as well as to lower the pressure in the biliary system and possibly reduce the incidence of anastomotic strictures or leaks by providing a mechanical support to the anastomosis [4]. Nonetheless, several systematic review and meta-analysis [4–6], have shown that the overall use of T-tube may not significantly modify the risk of either biliary leak or stricture after DDLT. Besides, the T-tube itself may cause biliary obstruction or leak and its removal may be associated with some degree of morbidity [4, 5]. Furthermore, bile deprivation due to external biliary drainage as well as the presence of an indwelling device in the biliary tree may be associated with other pathogenic effects, such as chronic inflammation, changes in intestinal microbiota, malabsorption and interruption of the enterohepatic cycle [7–9]. However, this latter evidence is so far based mainly on experimental data from animal models.

Many Centers have progressively changed their policies, limiting the use of T-tubes, but mainly relying on their own experience, in the absence of any standardized criteria. Therefore, the aim of the present survey was to assess the policies currently applied and the clinical rationale for the use of a T-tube in DDLT, among all the Italian LT Centers.

## Methods

A national survey was conducted using Google Forms (Google LLC, Mountain View, California, US) as the electronic data capture tool, from October to December 2020. The survey was sent by e-mail to the directors of all the Italian LT Centers performing adult LT. The e-mail content comprised a cover letter calling for participation and a hyperlink to the survey. The study was designed to understand the institutional profile, so only one senior surgeon from each center was invited to complete the questionnaire, with the agreement of all the members of the surgical team.

The survey was developed by the authors of the promoting LT Center (Liver-Kidney Transplant Unit, University Hospital of Udine) after the review the literature on the topic [4–10]. It consisted of 16 questions investigating the following aspects:

- LT center activity (with regard to 2019) [questions 1–5];
- institutional policy on use and management of T-tube in LT recipients [questions 6, 7, 9–13];
- prevalence of LT cases with T-tube in 2019 [question 9];
- institutional policy on administration of ursodesoxicholic acid (UDCA) after LT [question 14];
- potential pathogenic mechanisms associated with the use of T-tubes [questions 15, 16].

If the institutional policy excluded the use of T-tube in any case, a dedicated questionnaire was thus available for the responder [Section IIB instead of Section IIA], which included aspects (a), (d), (e) and (f) but further explored the institutional policy on biliary reconstruction [questions A, B, C]. The multiple response choices to questions in sections (c), (d) and (e) were elaborated on the basis of the available data from the relevant literature [4–10]; however, all questions had free text boxes for additional answers. The questionnaire was designed to be brief and completed by respondents in less than 10 min. The only questions which required a retrospective clinical data analysis were the annual LT volume and prevalence of T-tube use, in 2019. A copy of the original questionnaire is shown in Table 1. Data collection was considered completed when all the 20 Italian LT Centers had provided a response.

**Table 1:** A copy of the original questionnaire used in the survey

**Section I**	
1) How many adult LT procedures were performed in 2019 in your Center?	
2) Is a pediatric liver transplantation program active in your Center?	- Yes - No
3) Is a living-donor liver transplantation program active in you Center?	- Yes - No
4) Are grafts form donations after circulatory death (DCD) transplanted in your Center?	- Yes - No
5) Does the policy of your Center include the potential use of a T-tube in biliary reconstruction at LT?	- Yes (please go to section IIA) - No (please go to section IIB)
**Section IIA**	
6) Which indications are applied in your Center for the use of a T-tube in deceased donor adult liver transplantation? (more than one option is possible)	- No selective criteria, routine use - Split liver - Advanced donor age - Prolonged cold ischemia time - DCD grafts - Pre-donation acute liver injury (high transaminases levels) - Significant liver steatosis - Size discrepancy of bile ducts between donor and recipients - Small bile duct caliber - Re-transplantation - Delayed biliary reconstruction - High MELD recipient - Others—please specify
7) Has the policy of your Center changed in the last 5 years?	- No - Yes—please specify
8) In how many cases a T-tube was placed at transplantation in your Center, in 2019?	
9) Which are the criteria for T-tube clamping? (more than one option is possible)	- Negative trans-T-tube cholangiography - Low bilirubin serum levels - High bile output
10) In an uneventful LT case, when is T-tube routinely clamped? (postoperative day)	
11) Is the T-tube removed under endoscopic retrograde cholangiopancreatography (ERCP) control?	- Always - Never - Selectively: please specify
12) Is internal biliary stent, instead of T-tube, used in your Center?	- Never - Always - Occasionally - Selectively: please specify
13) In case of T-tube use, is there any protocol for bile replacement in your Center?	- No - Yes—please specify
14) Is ursodexocholic acid routinely administered to the recipient in the postoperative period?	- Always - Never - Selectively: please specify
15) Do you think the T-tube use may represent an hazard for postoperative morbidity other than mechanical biliary complications (leaks, strictures)?	- Yes - No
16) If yes, which pathogenic mechanisms may be implicated?	- Biliary inflammation due to an indwelling catheter - Bacterial colonization - Gut microbiota changes - Malabsorption - Interruption of the entero-hepatic bile-acid cycle - Other: please specify
**Section IIB**	
A) When did your Center opted for a no-T-tube policy?	- Less than 5 years ago - Between 5 and 10 years ago - More than 10 years ago
B) Which was the rationale that supported this policy change?	
C) Is any other mechanical support used in biliary reconstruction at LT?	- No, biliary reconstruction is always performed with duct-to-duct direct anastomosis or hepatico-jenjunostomy - Yes: please specify
14) Is ursodexocholic acid routinely administered to the recipient in the postoperative period?	- Always - Never - Selectively: please specify
15) Do you think the T-tube use may represent an hazard for postoperative morbidity other than mechanical biliary complications (leaks, strictures)?	- Yes - No
16) If yes, which pathogenic mechanisms may be implicated?	- Biliary inflammation due to an indwelling catheter - Bacterial colonization - Gut microbiota changes - Malabsorption - Interruption of the entero-hepatic bile-acid cycle - Other: please specify

Categorical variables were expressed by frequencies and percentages, while continuous variables were expressed by medians and interquartile ranges (IQR). Centers with an annual caseload over 70 cases were defined as high-volume Centers, according to a standard definition [11]. The association between LT center activity and institutional policies (T-tube and UDCA) was performed using Fisher’s test. Statistical significance was accepted for *p* < 0.05. Analyses were performed using Stata/SE 15.1 (Stata Corp LP, United States).

## Results

**Fig. 1:**
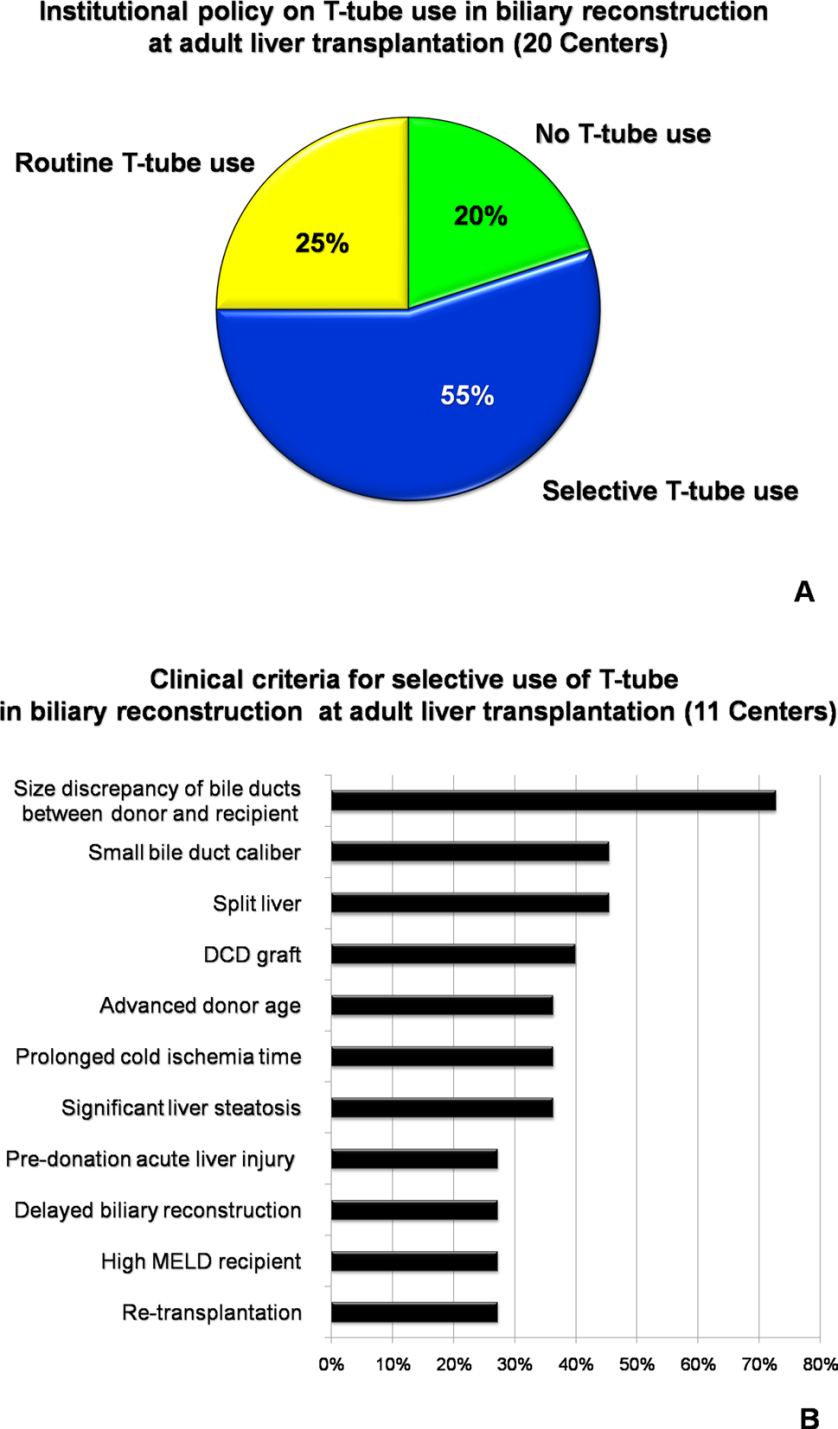
Graphic presentation of answers to question 5 (**a**) and 6 (**b**). *DCD* donation after circulatory death, *MELD* model for end stage liver disease

In 2019, a total of 1241 adult LT were performed in Italy by 20 Transplant Centers, with a median annual volume of 40 cases [33–91]. Pediatric and living-donor transplant programs were active in 4/20 (20%) and 9/20 (45%) Centers, respectively. Grafts from DCD were used in 12/20 (60%) Centers. The institutional policy completely excluded the use T-tube in 4/20 (20%) Centers, while 5/20 (25%) Centers used it routinely in all LT cases (Fig. 1a). The remaining 11/20 (55%) Centers applied a selective policy, based most frequently on criteria of technical complexity of biliary reconstruction (72.7%), followed by low-quality graft (63.6%) and high-risk recipient (36.4%). Figure 1b shows the specific selection criteria in details.

The four LT Centers which never used or discontinued the use of T-tubes, implemented such policy more than 10 years ago in 3/4 cases and between 5 and 10 years ago in the other one. In 3/4 Centers, the reported rationale was the lack of any significant evidence of advantage in controlling the risk of biliary complications, and the technique used in biliary reconstruction comprised direct duct-to-duct anastomosis or hepaticojejunostomy without any other mechanical support. One Center discontinued the use of T-tubes because they opted for internal absorbable biliary stents.

Among the 16 LT Centers where T-tubes were still in use, 7/16 (43.8%) reported a modification of their policy within the last five years, consisting of a switch from routine to selective use, the application of more stringent indications, or the use of smaller T-tubes. In 2019, LT cases with a T-tube represented more than 50% of the annual center caseload in 8/16 (50%) Centers, with only 5/16 (31.2%) using a T-tube in less than 25% of cases. An overall T-tube use in more than 50% of annual caseload was not associated with high-volume Center status [high-volume vs low-volume, 3/7 (42.9%) vs 5/13 (38.5%), *p* > 0.999], an active pediatric [active vs non-active, 1/4 (25%) vs 7/16 (43.7%), *p* = 0.619] or living-donor [active vs non-active, 3/9 (33.3%) vs 5/11 (45.5%), *p* = 0.670] transplant program, or use of DCD grafts [DCD grafts use vs no-use, 5/12 (41.6%) vs 3/8 (37.5%), *p* > 0.999].

**Fig. 2:**
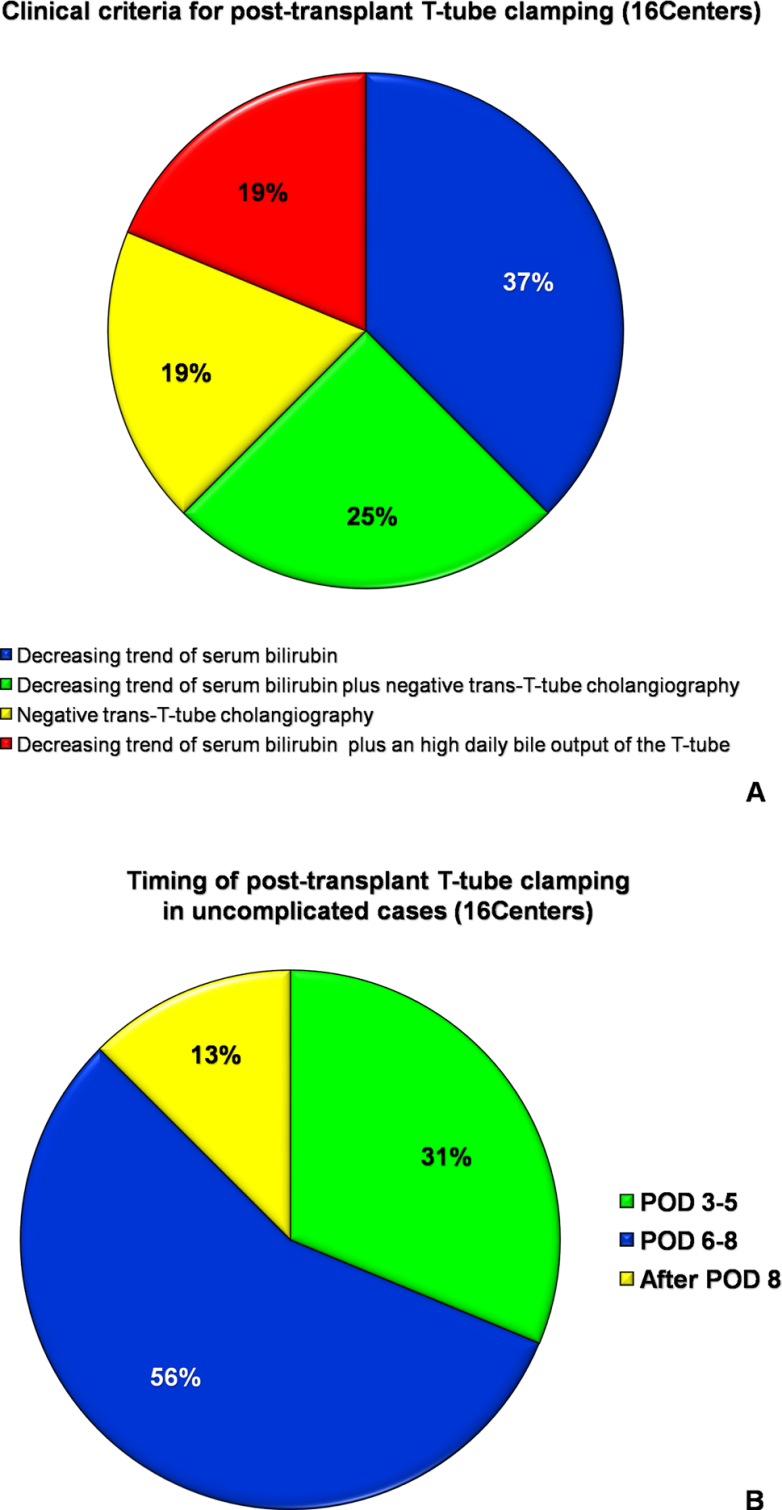
Graphic presentation of answers to question 9 (**a**) and 10 (**b**). *POD* postoperative day

The criteria for T-tube clamping included a decreasing trend of serum bilirubin in 6/16 (37.5%) Centers, a decreasing trend of serum bilirubin plus a negative trans-T-tube cholangiography in 4/16 (25%), a negative cholangiography in 3/16 (18.75%) and a decreasing trend of serum bilirubin plus an high daily bile output of the T-tube in 3/16 (18.75%) (Fig. 2a). In an uncomplicated postoperative course, the T-tube was routinely clamped on postoperative day (POD) 3–5 in 5/16 (31.3%) Centers, on POD 6–8 in 9/16 (56.3%), and after POD 8 in 2/16 (12.5%) (Fig. 2b). None of the Centers reported on the use of any protocol for bile replacement. On the other hand, 15/20 (75%) Centers routinely administered UDCA postoperatively, while only 1 (5%) Center used it selectively, in the presence of a small bile ducts and 4 (20%) Centers did not administer it routinely.

After a preliminary trans-T-tube cholangiography, the T-tube removal was programmed earlier than 3 months in 1/16 (6.25%) Center, at months 3 to 4 in 11/16 (68.8%) Centers and at month 6 or later in 4/16 (25%) Centers. The procedure was performed under endoscopic retrograde cholangiopancreatography (ERCP) control and with biliary stenting as a routine practice in 2/16 (12.5%) Centers; it was selectively programmed in the presence of biliary complication, biliary sludge or stones, or T-tube with large diameter, in 7/16 (43.7%) of Centers. Conversely, in the remaining 7/16 (43.75%) Centers, an ERCP was never used as technical or therapeutic aid during T-tube removal.

As an alternative to T-tube, the use of internal biliary stents was reported as sporadic by only 3/16 (18.7%) Centers.

Lastly, only 10/20 (50%) Centers considered the T-tube as a potential risk factor for complications other than biliary stricture/leak. In these cases, the suspected pathogenic mechanism comprised bacterial colonization (70%), malabsorption (70%), interruption of the entero-hepatic bile-acid cycle (50%), biliary inflammation due to an indwelling catheter (40%) and gut microbiota changes (40%). No other mechanisms were additionally reported.

## Discussion

In 2019, an international online survey investigating the technical practice in LT was conducted within the Eurotransplant, Swisstransplant, Scandiatransplant, and British Transplant Society networks [10]. To be noticed, none of the Italian Transplant Centers was enrolled in the survey. The use of a T-tube was reported by 14/42 (33.3%) of the responding Centers, either in an end-to-end or side-to-side biliary anastomosis. Its use was advocated to reduce biliary complications by mechanically supporting the biliary anastomosis in most cases (5/14, 57.1%), followed by a diagnostic purpose (35.7%) and a matter of institutional protocol and center routine (14.3%). As stated by the authors, the main message of the survey was that there is still a substantial heterogeneity and lack of best practices regarding the utilization of various surgical techniques in LT within Europe. Based on these data, we wanted to further investigate the standard practice of biliary reconstruction in the Italian LT setting, increasing the granularity of the information investigated and widening the clinical scenario to the post-transplant management. Biliary complications are still the most frequent surgical complications after LT, with a significant impact in terms of overall morbidity, quality of life and costs [4–6]. Therefore, a comprehensive analysis and critical discussion of the prevalent strategies in biliary reconstruction and T-tube use seemed a relevant aim to pursue. The first result to be outlined is that the prevalence of Centers using T-tubes in the present Italian survey was significantly higher than that of the European survey (80% vs 33.3%, *p* < 0.001). No association between the center caseload and the implemented surgical approach was noted in either surveys as well as the leading indication for T-tube was a technically high-risk biliary reconstruction in both of the surveys. In the present survey, the other main indication for T-tube use was to raise the postoperative surveillance of the graft functional recovery as well as of early surgical complications. Under this perspective, a low-quality graft, as determined by an advanced donor age, a prolonged cold ischemia time and a significant steatosis, were the major clinical concerns (4/11, 36.4% for each factor). Despite the evident effectiveness and reliability of bile output as a direct marker of liver graft function, in recent years, several non-invasive assessment methods have been developed, with comparable prognostic power [12–14]. The indocyanine green—plasma disappearance rate (ICG-PDR), either used as an intraoperative or early postoperative functional parameter, was demonstrated to be an effective predictor of early allograft dysfunction (EAD), primary non-function (PNF), and patient and graft survival [12, 13, 15]. Another functional test which has been developed and validated in liver resection surgery but recently applied also to LT, is the Liver Maximal Function Capacity (LiMax) which has been identified as an effective and early (within 24 h after LT) predictor of EAD/PNF [13, 14]. Beside functional tests, which are still under investigation and validation, the clinical practice has also been implemented with several clinical prognostic scores, such as the definition of EAD according to the criteria of Olthof et al. [16], the Model for Early Allograft Function (MEAF) score [17], the Liver Graft Assessment Following Transplantation (L-GrAFT) risk score [18], and more recently the Early Allograft Failure Simplified Estimation [EASE] score [19]. All these scores are based on routine laboratory tests, can be easily calculated, show a high predictive value for graft and patient survival, and have internally and externally been validated. Even in terms of biliary complications, the advances in imaging technology have nowadays provided diagnostic tools, such as contrast-enhanced magnetic resonance cholangiopancreatography (CEMRCP), which show a diagnostic performance that is almost comparable to trans-T-tube cholangiography [20].

T-tube may not only cause itself biliary obstruction or biliary leakage, but it also seems to sustain other pathogenic mechanisms resulting in non-mechanic biliary complications. In the present survey, only 50% of responding Centers acknowledged such risk, identifying bacterial colonization of the T-tube and malabsorption as the most probable underlying mechanisms. Indeed, several studies have reported on an increased risk of postoperative cholangitis or infected bilomas in association with the use of a T-tube [4, 21, 22]. However, the implicated pathogenic mechanism seems not to be limited to bacterial colonization of the T-tube. Bile acids regulate the gut microbiome structure by controlling gut bacteria composition and overgrowth by a selective antimicrobial effect, and protecting the epithelial barrier via farnesoid X receptor signaling [23]. Therefore, bile diversion through a T-tube in the early post-transplant period may result in gut microbiota changes, which have recently been shown to be associated with potential long-term inflammatory, metabolic and immune effects [24]. Moreover, hepatic bile acids have recently been identified as important mediators of early liver regeneration [7, 25]. Their depletion after major hepatectomy, by a bile salt-sequestering resin or external drainage in experimental models [7, 25], or by a T-tube in clinical studies [26], resulted in a significantly reduced liver regrowth. Moreover, a persistent pro-inflammatory state in the biliary tree or a postoperative bile leak has been also associated with an impaired liver regeneration in animal models [7, 26].

A greater agreement was found, among the Centers using a T-tube, on its postoperative management. The large majority of Centers clamped the T-tube on POD 6–7 and removed it 3–4 months post transplant, in uncomplicated LT cases. Some heterogeneity was found in the indications for clamping. However, a decreasing trend of serum bilirubin, either as single or composite parameter, represented the leading criteria (81%). Only 19% of Centers reported that a high bile output from the T-tube was also considered as a criteria for T-tube clamping, when associated with a decreasing trend of serum bilirubin, but none of the Centers reported on the use of any protocol of bile replacement in presence of an open T-tube. It was interesting to notice that the vast majority (75%) of centers administered unselectively UDCA to all LT recipients, although there are currently heterogeneous data supporting this clinical practice. UDCA solubilizes cholesterol from the surface and core of gallstones. Moreover, it seems to have a cytoprotective activity by decreasing the bile-acid/phospholipid ratio of the secreted bile, and by an anti-oxidant, anti-apoptotic and membrane-stabilizing effect [27]. A recent meta-analysis of two retrospective studies and one randomized controlled trial (RCT) have demonstrated that UDCA did significantly reduce the risk of post-LT biliary stones and sludge but did not have any significant impact on the risk of biliary strictures [27]. On the other hand, a meta-analysis of four high-quality RCTs has shown that UDCA, as an adjuvant treatment, was not able to prevent acute cellular rejection or steroid-resistant rejection after LT [28].

Very limited data are available on the best timing for T-tube removal, but it is usually accepted that a T-tube should remain in place for a minimum of 3–6 months after LT to allow for sufficient time for biliary fistulous formation [29]. However, there are some reports that have identified a T-tube removal earlier than 6 months in DDLT [30] as an independent risk factor for biliary stricture, and earlier than 8 months in LDLT [29] as a risk factor for post-removal biliary leakage. Overall, the result of the present survey was in line with the prevalent practice. The procedure of T-tube removal carries the risk of biliary leakage, with a reported prevalence of 20–30%. The majority of cases is a self-limiting complication which only requires clinical surveillance, but in rare cases, it may worsen to biliary peritonitis, thus requiring invasive treatments [29, 31]. Under this perspective, this procedure may be undertaken under ERCP control, with placement of a stent to cover the biliary defect. However, such benefit should be balanced with the inherent morbidity risk of ERCP [32]. Thus, a selective approach that limits ERCP use only to cases with evidence of biliary anomalies (stricture, sludge, stones) on pre-removal cholangiography may appear the most appropriate.

This study presents several limitations: the data required to complete the survey were retrospectively analyzed; the specific technique used in biliary reconstruction and the Center's prevalence of biliary complications were not evaluated, since the primary endpoint of the study was to investigate the institutional policies on T-tube use, rather than assessing the impact of T-tube on post-LT outcomes. Nonetheless, we believe that the present results may actually promote further investigations, aimed at exploring the impact of the diversified Center policies on the risk of post-transplant complications.

## Conclusion

In the present survey, the prevalence of T-tube use among the Italian LT Centers is still relatively high, compared to the European trend, and the potential detrimental effects of T-tubes, beyond biliary stricture/leakage, are probably underestimated. We believe that nowadays the use of a T-tube as a surveillance tool of graft functional recovery may no longer be necessary in clinical practice, thanks to the advances in imaging technology and clinical tests. On the other hand, complex biliary reconstructions may still be advantaged by the use of a mechanical tutor. Strategies to reduce the potential functional morbidity of T-tube should further be explored and implemented in clinical practice. Nonetheless, the European trend as well as the experience of the Italian Centers that have completely discontinued the use of T-tubes, clearly demonstrates that T-tube-free biliary anastomosis is feasible and should be considered as realistic and probably warranted in clinical practice.

## Data Availability

The datasets generated during and/or analysed during the current study are available from the corresponding author on reasonable request.
